# Model-based analysis of stop-signal data reveals robust neural and clinical correlates of evidence accumulation but not inhibition

**DOI:** 10.1038/s41386-026-02401-6

**Published:** 2026-04-22

**Authors:** Yihe Weng, Rory Boyle, Chi Tak Lee, Declan Quinn, Clodagh Earley, Maike Splittgerber, Lili Zhang, Luisa Franzen, Tobias Banaschewski, Arun L. W. Bokde, Sylvane Desrivières, Herta Flor, Antoine Grigis, Hugh Garavan, Penny Gowland, Andreas Heinz, Rüdiger Brühl, Jean-Luc Martinot, Marie-Laure Paillère Martinot, Eric Artiges, Jane McGrath, Frauke Nees, Dimitri Papadopoulos Orfanos, Luise Poustka, Nathalie Holz, Sarah Hohmann, Michael N. Smolka, Nilakshi Vaidya, Gunter Schumann, Henrik Walter, Alexander Weigard, Robert Whelan, IMAGEN Consortium

**Affiliations:** 1 School of Psychology and Global Brain Health Institute, Trinity College Dublin, Dublin, Ireland.; 2 School of Computing, Dublin City University, Dublin, Ireland.; 3 Faculty of Psychology and Neuroscience, Maastricht University, Maastricht, Netherlands.; 4 Department of Child and Adolescent Psychiatry and Psychotherapy, Central Institute of Mental Health, Medical Faculty Mannheim, Heidelberg University, Square J5, 68159 Mannheim, Germany.; 5 German Center for Mental Health (DZPG), Partner Site Mannheim-Heidelberg-Ulm, Heidelberg, Germany.; 6 Discipline of Psychiatry, School of Medicine and Trinity College Institute of Neuroscience, Trinity College Dublin, Dublin, Ireland.; 7 Social, Genetic and Developmental Psychiatry Centre, Institute of Psychiatry, Psychology & Neuroscience, King’s College London, London, UK.; 8 Institute of Cognitive and Clinical Neuroscience, Central Institute of Mental Health, Medical Faculty Mannheim, Heidelberg University, Square J5, Mannheim, Germany.; 9 Department of Psychology, School of Social Sciences, University of Mannheim, 68131 Mannheim, Germany.; 10 NeuroSpin, CEA, Université Paris-Saclay, F-91191 Gif-sur-Yvette, France.; 11 Departments of Psychiatry and Psychology, University of Vermont, 05405 Burlington, Vermont, USA.; 12 Sir Peter Mansfield Imaging Centre School of Physics and Astronomy, University of Nottingham, University Park, Nottingham, UK.; 13 Department of Psychiatry and Psychotherapy, University of Tübingen, Tübingen, Germany.; 14 German Center for Mental Health (DZPG), Site Tübingen, Germany.; 15 Department of Psychiatry and Psychotherapy, Technische Universität Dresden, Dresden, Germany.; 16 Physikalisch-Technische Bundesanstalt (PTB), Braunschweig and Berlin, Berlin, Germany.; 17 INSERM U1299 “Developmental Trajectories in Psychiatry & Addictology”; University Paris-Saclay, Ecole Normale Supérieure Paris-Saclay, Mathematics department, Centre Borelli CNRS UMR9010, Gif-sur-Yvette, France.; 18 Department of Psychiatry, LabD-Psy, Barthélémy Durand Hospital, Etampes, France.; 19 AP-HP. Sorbonne Université, Department of Child and Adolescent Psychiatry, Pitié-Salpêtrière Hospital, Paris, France.; 20 Institute of Medical Psychology, Ludwig-Maximilians-Universität (LMU) in Munich, Munich, Germany.; 21 Department of Child and Adolescent Psychiatry, Center for Psychosocial Medicine,, University Hospital Heidelberg, Heidelberg, Germany.; 22 Department of Child and Adolescent Psychiatry, Psychotherapy and Psychosomatics, University Medical Centre Hamburg-Eppendorf, Hamburg, Germany.; 23 Centre for Population Neuroscience and Stratified Medicine (PONS), Department of Psychiatry and Psychotherapy, Charité Universitätsmedizin Berlin, Berlin, Germany.; 24 Centre for Population Neuroscience and Stratified Medicine (PONS), Institute for Science and Technology of Brain-Inspired Intelligence and National Centre for Neurological Disorders, Huashan Hospital, Fudan University, Shanghai, China.; 25 German Center for Mental Health (DZPG), Site Berlin-, Potsdam, Germany.; 26 Department of Psychiatry and Psychotherapy CCM, Charité – Universitätsmedizin Berlin, corporate member of Freie Universität Berlin, Humboldt-Universität zu Berlin, and Berlin Institute of Health, Berlin, Germany.; 27 Department of Psychiatry and Addiction Center, University of Michigan, 4250 Plymouth Rd, Ann Arbor, MI 48109, USA.; 28 These authors contributed equally: Alexander Weigard, Robert Whelan.

## Abstract

Poor inhibitory control and decision-making are often considered as risks for substance use and other adverse psychiatric outcomes. The Stop-Signal Task (SST) is a widely used protocol, from which inhibitory control is indexed by stop signal reaction time (SSRT). However, heretofore models of SSRT may be too simplistic to capture complex processes underlying task performance. In contrast, the Racing Diffusion Ex-Gaussian ABCD (RDEX-ABCD) model provides a more mechanistic framework, capturing both inhibitory control and task-general decision-making processes during the SST. Here, we applied the RDEX-ABCD model to SST data from the IMAGEN cohort (n > 1000) at ages 19 and 23, and examined model parameters in relation to substance use via Elastic Net regression. Connectome-based predictive modeling was then performed to identify brain networks predicting parameters, and the association between these networks and substance use was examined. We found that parameters indexing inhibitory control had no associations with substance use and were only weakly associated with brain connectivity. In contrast, parameters reflecting general decision-making processes – such as efficiency of evidence accumulation, decision threshold (response caution), probability of go failure – and their associated brain activity were significant predictors of cannabis and cigarette use. These findings suggested that efficiency of evidence accumulation, a neurocognitive mechanism that facilitates adaptive decision making across many contexts, emerged as a robust predictor of substance use vulnerability. Overall, general decision-making mechanisms may act as more reliable indicators of vulnerability to substance use than the conventional inhibitory control measures.

## INTRODUCTION

Inhibitory control is a core cognitive process implicated in several psychiatric disorders [1, 2], such as substance use disorders or attention deficit hyperactivity disorder (ADHD). As a latent construct, inhibitory control is often inferred from task performance. The Stop-Signal task (SST) requires individuals to complete a choice response time task; on a subset of trials, infrequent and unpredictable stop signals that the prepotent response should be withheld. The stop signal reaction time (SSRT) is a latent measure of individual differences calculated from SST performance. The measurement of SSRT is often based on the assumptions of the *independent race model* [3], which posits that a latent “stop” process races the “go” response process, with the winner determining whether the response is successfully inhibited. This model remains the most prevalent theoretical framework for SSRT estimation [4]. However, this model is too simplistic to capture the full array of complex cognitive processes involved in performance of the SST [5].

Recent advances in computational modeling enable the use of *cognitive process models* with precise assumptions about the mechanistic processes contributing to behavior, offering a comprehensive description of SST performance. The ex-Gaussian race model [6, 7] describes Go reaction time (RT) and SSRT distributions using parametric assumptions about mechanisms underlying the latency and variability of the go and stop processes [4, 5]. Racing diffusion models go further by positing that the go and stop processes both involve accumulation of noisy evidence from the environment until that evidence crosses a critical threshold for either initiating a go choice response or stopping the response [8]. As racing diffusion models provide a more detailed description of processes involved in the SST than ex-Gaussian race models but can have problematic parameter recovery properties [9], a hybrid racing diffusion ex-Gaussian model (RDEX) has been proposed [10].

Critically, cognitive process models have begun to provide novel insights about inhibitory performance in clinical groups. Application of these models to case-control samples in ADHD [11] and schizophrenia [12] indicate that poorer inhibitory performance in both disorders is likely related to broader problems with inattentive and inefficient processing rather than specific inhibition deficits. Although case-control studies demonstrate the clinical relevance of cognitive process models, such models have yet to be applied in broader population samples to better characterize the neural and clinical correlates of individual differences in mechanisms of SST performance. Further, no prior studies have used these SST models to characterize neurocognitive risk factors for substance use, a behavior that is widely thought to be linked to poorer inhibitory control [2, 13].

**Fig. 1:**
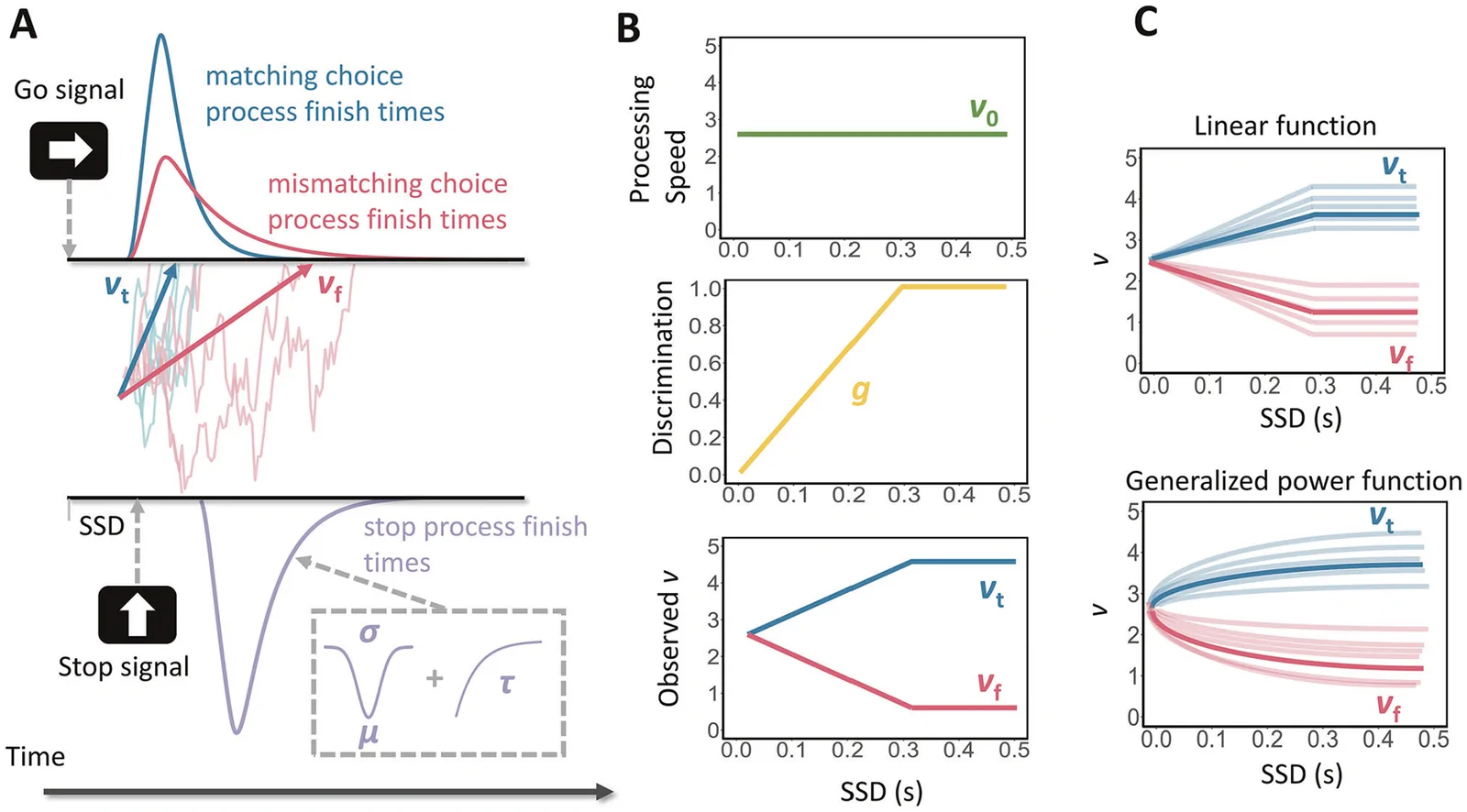
RDEX-ABCD model of the Stop-Signal task. **A** The “hybrid” racing-diffusion ex-Gaussian (RDEX) model framework combining an evidence-accumulation model for the go process with an ex-Gaussian model for the stop process. In this framework, Go reaction times (RT) results from a competition between accumulators that collect noisy evidence for both the choice matching the stimulus and the choice mismatching it (e.g., a right-facing arrow). These accumulators operate at average rates of *v*_t_ and *v*_f_, respectively, and the process concludes when one of the accumulators reaches a predefined response threshold. The stop process is the Gaussian distribution characterized by a mean (*μ*) and standard deviation (*σ*), convolved with an exponential distribution with a mean of *τ*. **B** The RDEX-ABCD model accounts for the impact of context independence violations on Stop trials by introducing a perceptual growth function. In this function, evidence signals for both the matching and mismatching accumulators on Stop trials with a given stop signal delay (SSD) are composed of two components: a processing speed component that influences evidence accumulation equally for both choices and a discrimination component that favors the choice aligned with the presented stimulus. The processing speed is determined by parameter *v*_0_ and remains constant across all SSDs. At an SSD of 0 s, the discrimination component is absent (equal to 0) since the choice stimulus has not been presented yet. However, this component increases linearly at the same rate *g* for both the matching and mismatching accumulators as SSD lengthens until it reaches the levels of *v*_t_ and *v*_f_, as observed in Go trials. Consequently, at an SSD of 0 s, the rates for matching and mismatching choices are identical, but as SSD increases, these rates diverge gradually until they match their respective Go trial levels. Panel **B** illustrates the linear growth form. **C** Empirical growth patterns of matching (blue lines increasing from SSD = 0) and mismatching (red lines decreasing from SSD = 0) go process accumulator rates by SSD for the sample average parameter estimates (thick lines) and for parameter estimates from several randomly drawn participants (thin lines) to illustrate individual variability. In panel **C**, the top subpanel shows the linear perceptual growth function, and the bottom subpanel shows the non-linear (power) growth function (determined via model comparison). This figure is adapted and modified from [16] (Figure is licensed under CC BY-NC-ND 4.0).

The IMAGEN study is a large longitudinal neuroimaging study of community-recruited European youth [14], spanning mid-adolescence to young adulthood, with extensive characterization of substance use. Hence, application of cognitive process models to IMAGEN provides a key opportunity to characterize the neural correlates of the array of mechanistic processes contributing to SST performance and link these processes to substance use. IMAGEN includes an SST with a design identical to the SST used in the ABCD study [14, 15]. This allows the RDEX-ABCD model [16], which was recently developed to address context independence violations in the ABCD task [17, 18], to be applied to IMAGEN (See Methods and Fig. 1 for details). Application of RDEX-ABCD within IMAGEN provides a portable framework for deriving mechanistically interpretable latent decision-process parameters, linking them to neural circuitry, and using them to make predictions about future substance use in the ABCD cohort once youth in ABCD mature. Critically, the relevance of this work also extends well beyond IMAGEN and ABCD; these large, well-powered cohorts yield stable effect estimates that can serve as a benchmark for replication in independent samples.

Two processes measured by RDEX-ABCD are of particular relevance to understanding individual differences in substance use. First, *efficiency of evidence accumulation* (EEA) for decision making processes is a neurocognitive risk factor for externalizing behavior and substance use [19–21]. Second, lower values of the *decision threshold* for choice processes, reflecting individuals’ response caution, have been linked with substance use behaviors [14, 22, 23]. However, these previous studies were limited by small samples, low power, and minimal consideration of the neuroimaging correlates. Characterizing the neural correlates of these processes allows us to link computational measures to brain function, improving understanding of the biological mechanisms contributing to substance use risk.

Emerging neuroimaging work indicated that evidence accumulation was supported by frontoparietal network [24–26] and decision threshold is associated with fronto-basal ganglia network [26, 27]. However, prior studies have largely relied on region-based activation analyses, limiting inferences about how distributed, whole-brain functional networks support individual differences in these cognitive processes. Therefore, we adopted connectome-based predictive modeling (CPM) [28, 29], a well-validated data-driven machine learning approach, to identify brain networks that predict behavioral performance on the SST based on whole-brain functional connectivity.

In this study, we used the IMAGEN sample (*n* > 1000) to assess how mechanistic processes captured by the RDEX-ABCD model relate to whole-brain functional connectivity (derived from a previous study [30]) and substance use. A prior report using the same dataset focused on only Go-trial RT variability as a putative sustained-attention index and examined longitudinal associations with substance use [30]. Here, we extend this work by applying RDEX-ABCD to both Go and Stop trials at ages 19 and 23 to dissociate latent decision processes, and by machine learning methods (Elastic Net regularized regression and CPM) to identify robust links between these processes (and their neural correlates) and substance use. We hypothesized that computational parameters would show stronger and more specific associations with neural connectivity and substance use than SSRT from the standard integration method. Based on previous findings [24, 26], we expected EEA-related network activity to primarily involve the frontoparietal network, whereas networks associated with decision threshold would involve fronto-basal ganglia circuitry; additionally, higher EEA and lower decision threshold were expected to be associated with greater substance use.

## METHODS AND MATERIALS

### Participants

This study used data from IMAGEN at ages 19 and 23. Written and informed consent was obtained from all participants by the IMAGEN consortium, and the study was approved by the institutional ethics committees of all participating sites in accordance with the Declaration of Helsinki (see the Supplementary Materials for site-specific approvals). Data from age 14 were excluded because the stimulus delivery program at this age did not record response and RTs for trials with an SSD of 0, rendering modeling impossible. Participants were excluded based on criteria outlined by Weigard et al. [16]; if their behavioral data did not align with race model assumptions (See Supplementary Materials for details). The final sample for model estimation included 1256 and 1089 participants at ages 19 and 23, respectively. For fMRI prediction analysis, participants with excessive head motion (mean framewise displacement>0.5 mm) were additionally excluded (*n* = 1202/1045 at age 19/23). Details on fMRI acquisition, and preprocessing details are available in a previous study [30].

### Stop-signal task

The SST required participants to respond to a Go signal —an arrow pointing left or right— the corresponding left or right button on Go trials, while withholding their response if the Go signal was unpredictably followed by a Stop signal (an upward-pointing arrow) on Stop trials (see [30]). To adjust task difficulty dynamically, a tracking algorithm on the delay was used between the Go signal and Stop signal (stop-signal delay, 250–900 ms in 50 ms increments) [4], to produce ~50% successful inhibition trials. This task included 300 Go trials and 60 Stop trials with variable delays.

### Timeline followback

The Timeline Followback, a calendar-based interview with established strong reliability and validity [31], was used to evaluate prior alcohol, drugs, and smoking consumption over the past 30 days. Distributions of the frequency measures (including a subset of high-frequency users) are shown in Fig. S1, and the corresponding items are provided in Table S1.

### RDEX-ABCD model

The RDEX-ABCD model combines an evidence-accumulation model of the go process with an ex-Gaussian model of the stop process (Fig. 1A). This model assumes that Go RTs and choice accuracy arise from a race between accumulators gathering noisy evidence for the matching and mismatching choices, with match rate (*v*_t_) and mismatch rate (*v*_f_), until a response threshold (*B*) is reached. Here, ‘matching choice’ refers to selecting the response corresponding to the direction of the Go stimulus, whereas ‘mismatching choice’ refers to selecting the opposite (incorrect) response. Stop process finishing times are assumed to follow an ex-Gaussian distribution, defined by Gaussian mean (*μ*) and standard deviation (*σ*) parameters, convolved with an exponential tail (*τ*), which effectively captures both the central tendency and skew of RT data [32]. The model includes the probability of go failure (*P*_gf_) which captures omissions or failures to respond to the Go signal on Go trials, and the probability of trigger failure (*P*_tf_), which reflects failures to initiate the inhibitory process following a Stop signal on Stop trials due to attentional lapses [11, 33, 34]. The RDEX-ABCD model addresses violations of context independence by introducing a perceptual growth function [16]. This function characterizes the relationship between stop-signal delay (SSD) and Go-stimulus processing, modeled as either a linear or non-linear function (see Supplementary Materials and Fig. 1B).

### RDEX-ABCD model evaluation and estimation

We tested four variants of the original RDEX-ABCD model to evaluate cognitive processes in IMAGEN. Two features were assessed: (1) whether including *P*_tf_ improved model fit, and (2) whether a generalized power function better captured growth in choice evidence quality than the linear form (Fig. 1C). Model complexity and number of parameters are detailed in Table S2. Model performance was compared using the Deviance Information Criterion [35] and Bayesian Predictive Information Criterion [36], which reward goodness-of-fit while penalizing unnecessary complexity; lower values indicate better fit.

Models were estimated using the RDEX-ABCD code (available at https://osf.io/2h8a7/) within the Dynamic Models of Choice (DMC) framework (a free set of R functions for Bayesian estimation and simulation of evidence accumulation and stop-signal task models [37]). Individual-level Bayesian estimation employed a differential-evolution Markov chain Monte Carlo algorithm [38] to sample from the posterior distribution, which is particularly suitable for evidence-accumulation models because it allows for more efficient sampling when model parameters are correlated. For Bayesian estimation, an initial exploratory analysis used the informative priors derived from the ABCD dataset [16], whereas broad, uninformative priors were adopted for the main analysis (Table S3). To ensure robustness of the parameter estimates, we also performed parameter recovery, with full details of model estimation and recovery provided in the Supplementary Materials.

### EEA and SSRT calculation

Two higher-order parameters – EEA and SSRT_RDEX_ – were derived from the model. EEA was computed by subtracting mismatching rates from matching rates. SSRT_RDEX_ was computed by simulating finishing time from ex-Gaussian posterior distributions [16], and SSRT_INT_ was estimated using the integration method [4, 39]. Further details on SSRTs estimation are provided in Supplementary Materials.

### Relationship between substance use and RDEX-ABCD model parameters

#### Exploratory factor analysis on timeline followback.

Exploratory factor analysis was performed on ten selected items from Timeline Followback, yielding three common factors scores related to alcohol, cigarette and cannabis (Cig+CB), and drug use. According to the rotated factor loadings, the factors were labeled as (i) *alcohol*, (ii) *Cig* + *CB*, and (iii) *drug* (including cocaine, ecstasy, and ketamine) use. Detailed information is provided in Supplementary Materials and in a previous study [30]. These factor scores were subsequently used to measure substance use in the analysis.

#### Elastic net regression.

To assess the relationship between RDEX-ABCD parameters and substance use, we applied Elastic Net regularized regression, which is a multivariable approach that combines the L1 (LASSO) and L2 (ridge) penalties. Here, 11 RDEX-ABCD parameters compromising the optimal model (See Results) and covariates (age, sex, and sites) served as predictor variables, and the substance use score (e.g., Cig+CB) derived from Timeline Followback as targets. We applied a ten-fold nested repeated cross-validation using the *glmnet* [40] and *nestedcv* packages [41] in Rstudio and R (v4.3.1). In the nested cross-validation procedure, the inner loop optimized the hyperparameters α and λ via a grid search [42], selecting the set with the lowest mean RMSE. These optimal hyperparameters were then applied in the corresponding outer loop to evaluate performance. Performance was averaged across outer folds to yield final RMSE and R^2^. A final model was fitted on the whole data using the median hyperparameters from outer folds to estimate feature-wise beta values. To assess the stability of model performance and feature weights, the Elastic Net regression was repeated 100 times. From these repetitions, we calculated the mean model performance (RMSE and R^2^), the mean regression coefficient (β) and selection frequency for each predictor. To evaluate the model’s statistical significance, a permutation test (1000) iterations generated a null distribution of model performance. The mean model performance from the 100 repetitions was then compared against this null distribution using a significance threshold of *P* < 0.05. The Elastic Net regression was performed to assess the relationship between RDEX-ABCD parameters and substance use, including Cig+CB, alcohol use, and drug use. A schematic (Fig. S2) and further methodological details are provided in the Supplementary Materials.

#### CPM prediction.

CPM was performed to explore individual differences in brain connectivity related to behavioral variables [28, 29]. Two whole-brain generalized psychophysiological interaction matrices [43] — for Successful Stop or Go trials — were generated using the Shen atlas (See Supplementary Materials and our previous study [30] for details).

The CPM was implemented using ten-fold cross-validation. First, functional connection (i.e., edges) in matrices associated with behavioral variables (i.e., model parameters) were selected using a threshold of *P* < 0.01, controlling for age, sex, site, and head motion in the training set. Edges positively/negatively correlated with parameters were regarded as positive/negative predictive networks. Network strength was calculated by summing the edge weights, with the combined network defined as positive minus negative strength. Linear predictive models were constructed between network strength and model parameters in the training set, with age, sex, site and mean framewise displacement (head motion) included as covariates. In the test set, network strengths were input into the predictive model, along with covariates, to predict behavioral variables. To avoid over-fitting to a random ten-fold split [44], CPMs were repeated 100 times, yielding averaged model performance. Model significance was assessed using a permutation test (1000 iterations), in which behavioral variables were randomly shuffled across participants while keeping connectivity and covariates data fixed. The full CPM procedure was rerun for each permutation to generate a null distribution of predictive performance for significance testing [45].

The Elastic Net regression identified match rate (*v*_t_), probability of go failure (*P*_gf_), and response threshold (*B*) as the strongest predictors of cigarette and cannabis use (Cig+CB; see Results). Therefore, we assessed the predictive networks associated with these three cognitive processes and their relationship with Cig+CB. Additionally, the higher-order parameter EEA (the difference between match and mismatch rates), captures overall efficiency of evidence accumulation across both types of choices and has been linked to substance use [19, 20]. Accordingly, CPM analysis focused on EEA rather than *v*_t_ to identify brain networks associated with the efficiency of evidence accumulation, as well as on *P*_gf_ and *B*. Additional CPM analyses were performed to predict SSRT_RDEX_ and SSRT_INT_. Because EEA, *B*, and *P*_gf_ reflect the go process, connectivity matrices from Go trials were used to predict these parameters. Conversely, since SSRT reflects the stop process, connectivity matrices from Successful Stop trials were used to predict SSRT_REDX_ and SSRT_INT_. We focused on the predictive performance of the combined network.

#### Correlation between network strength and substance use.

Correlations between network strength of parameters and Cig+CB were assessed. Spearman correlations were calculated between residual network strength of parameters (controlling for age, sex, site, and head motion) and residual Cig+CB score (controlling for age, sex, and site).

#### Statistical analysis.

To evaluate consistency over time, correlations of model parameters, and predictive network strength between two time-points were calculated.

## RESULTS

### Model fit

Demographic information for participants included in RDEX-ABCD analyses is shown in Table 1. The model estimation included 1256 participants aged 19 and 1089 participants aged 23.

The model comparison results, as indicated by the Deviance Information Criterion and Bayesian Predictive Information Criterion values, demonstrated that the power function model without *P*_tf_ was the winning model for ages 19 and 23 (Table S2). The lack of evidence for the *P*_tf_ parameter suggests that occasional failures to initiate the inhibitory process (i.e., due to attentional lapses) have a reduced impact in this age group than in younger adolescents from ABCD study. Evidence favoring power function over linear growth further indicates that the relationship between SSD duration and Go-signal processing (e.g., the perceptual growth function) follows a non-linear trajectory in these older participants. The final model included 11 core parameters (see Table S4 for mean values).

The posterior predictive checks (Fig. S3) confirmed that the RDEX-ABCD model provided an adequate description of the real data for the IMAGEN study. Parameter recovery analysis (Figs. S4, S5) indicated that the model parameters could be reliably estimated. Together, these results indicate that the RDEX-ABCD model reliably estimates parameters and adequately captures the observed data.

### Parameter correlation over time

**Fig. 2:**
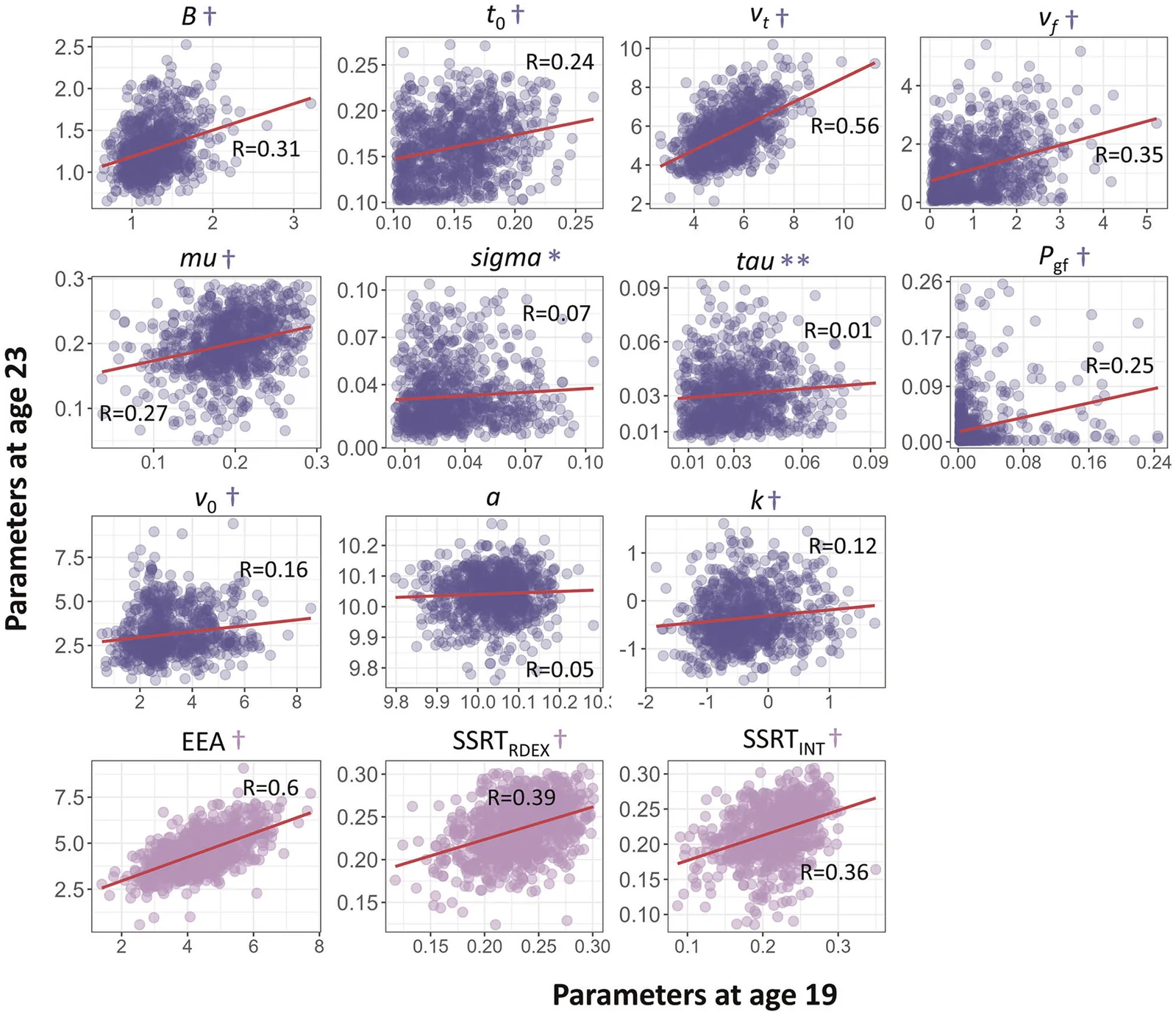
Correlation of RDEX-ABCD parameters between ages 19 and 23. The first three rows are original parameters yielded from the RDEX-ABCD model. The last row represents the parameter estimated from the model parameters. *, *P* < 0.05; **, *P* < 0.01; †, *P* < 0.001.

Figure 2 displays correlations of model parameters between ages 19 and 23. Correlations for SSRT derived from the RDEX-ABCD model (SSRT_RDEX_) and integration method (SSRT_INT_) did not differ significantly (r’s=0.39 vs. 0.36, *P* = 0.465).

### RDEX-ABCD model parameters and substance use

**Fig. 3:**
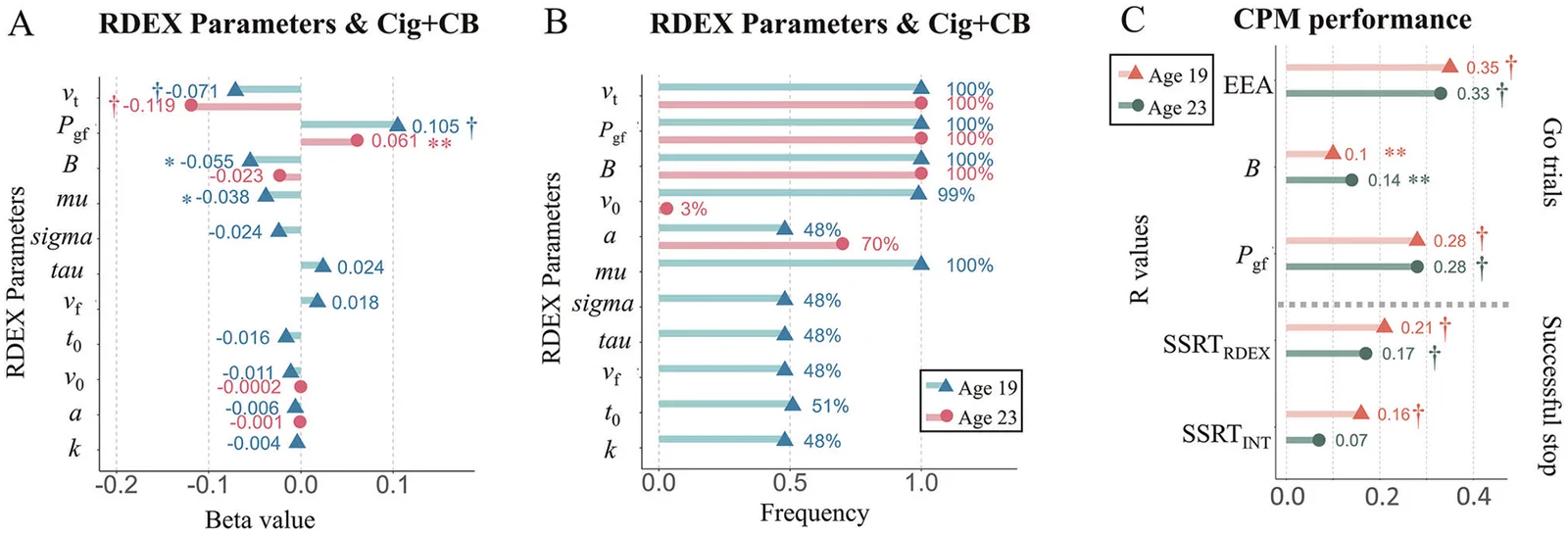
Prediction of Cig+CB scores using RDEX parameters. **A** Mean beta value for each parameter predicting Cig+CB score in Elastic Net regression model at ages 19 and 23. **B** Frequency of each parameter predicting Cig+CB in 100 iterations of Elastic Net regression model. **C** Predictive performances of connectome-based predictive modeling (CPM). Cig+CB, Cigarette and cannabis use. EEA, efficiency of evidence accumulation; **B** decision threshold; P_gf_, probability of go failure. SSRT_RDXE_, stop signal reaction time derived from RDEX-ABCD; SSRT_INT_, stop signal reaction time derived from integration method; *, *P* **<** 0.05; **, *P* **<** 0.01; †, *P* **<** 0.001.

Elastic Net regression was conducted at each timepoint to assess whether RDEX-ABCD parameters predicted factor score derived from Timeline Followback. Model metrics (RMSE and R^2^) for Cig +CB were significantly better than null models generated via random-label permutation (*P’s* < 0.001) at ages 19 and 23. Figure 3 displays the mean coefficients and selection frequencies of RDEX-ABCD parameters across 100 iterations of Elastic Net regression. At age 19, all parameters correlated with Cig+CB, with the top three feature weights being *P*_gf_, *v*_t_, and *B* (β’s = 0.105, −0.071, −0.055, respectively); remaining parameter values had smaller effect (| β’s | < 0.04). At age 23, five parameters correlated with Cig+CB, with *v*_t_, *P*_gf_ and *B* as the top three (β’s = −0.119, 0.061, −0.023, respectively); These three were selected for all 100 Elastic Net iterations at both ages.

There were no significant correlations between any model parameters and alcohol/other drug use at ages 19 and 23 (all *P* > 0.05, Fig. S2).

### CPM results

Figure 3C shows the CPM results. Combined networks significantly predicted EEA, *B*, and *P*_gf_ at age 19 (r’s=0.37, 0.10, 0.28, respectively, *P’s* < 0.01) and at age 23 (r’s=0.33, 0.14, 0.28, respectively, *P’s* < 0.01). The combined network predicted SSRT_RDEX_ (*r* = 0.21, *P* < 0.001) and SSRT_INT_ (*r* = 0.16, *P*’s < 0.001): these r values were not significantly different from each other (*P* = 0.30) at age 19. At age 23, the network predicted SSRT_RDEX_ (*r* = 0.17, *P* < 0.001) but not SSRT_INT_ (*r* = 0.07, *P* = 0.077): these *r* values were significantly different (*P* = 0.019). The above results were validated using a ten-fold cross-validation; similar results were obtained using five-fold cross-validation and leave-site-out cross-validation (Table S5).

**Fig. 4:**
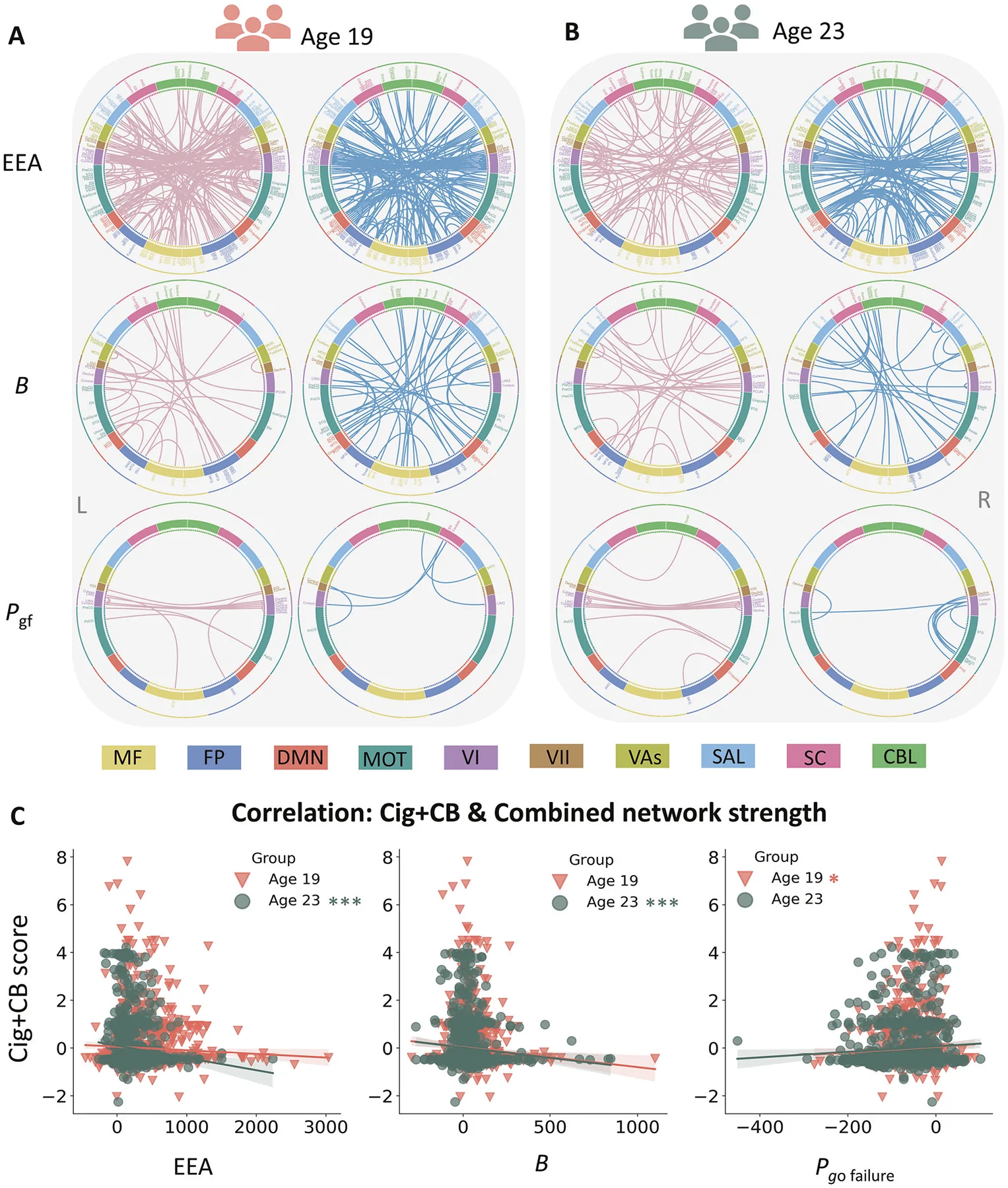
Connectome patterns and associations with substance use. **A** Connectome pattern of predictive networks at age 19. **B** Connectome pattern of predictive networks at age 23. Pink and blue color represent positive and negative networks separately. **C** Correlation between cigarette and cannabis use (Cig+CB) and combined network strength derived from Go trials. MF Medial frontal, FP Frontoparietal, DMN Default mode, MOT Motor, VI Visual I, VII Visual II, Vas Visual association, SAL Salience, SC Subcortical, CBL Cerebellar. R/L, right/left hemisphere. *, *P* < 0.05; **, *P* < 0.01; ***, *P* < 0.001.

The brain networks predicting EEA, *B and P*_gf_ at ages 19 and 23 are shown in Fig. 4A, B, with the top contributing connections and node degree maps provided in Figs. S6–8. The brain networks predicting SSRT_RDEX_ and SSRT_INT_ at ages 19 and 23 are shown in Fig. S9, with the top contributing connections and node degree maps presented in Figs. S10–S11.

### Correlation between network strength at ages 19 and 23

The network strength of EEA (*R* = 0.60, *P* = 3.02E-79), *B* (*R* = 0.30, *P* = 6.58E-18), *P*_gf_ (*R* = 0.25, *P* = 2.64E-12), SSRT_RDEX_ (*R* = 0.42, *P* = 3.50E-34) and SSRT_INT_ (*R* = 0.25, *P* = 1.71E-11) were correlated between ages 19 and 23 (Table S6).

### Correlation between network strength and Cig+CB

At age 19, only the *P*_gf_ network strength correlated with Cig+CB (Rho=0.07, *P* = 0.016), whereas EEA and *B* network strengths showed no significant correlations (Rho = −0.04, *P* = 0.226; and Rho = −0.06, *P* = 0.060; Fig. 4C). At age 23, the EEA and *B* network strength – but not *P*_gf_ – correlated with Cig+CB (Rho = −0.12, *P* = 2.57E-04; Rho = −0.11, *P* = 4.99E-04; and Rho=0.06, *P* = 0.058 respectively). No significant correlations were observed for the SSRT_RDEX_ or SSRT_INT_ network strength and Cig+CB (*P*’s > 0.05). *P* values were corrected using false discovery rate correction (*q* < 0.05). Detailed results are shown in Table S7.

## DISCUSSION

The RDEX-ABCD model was applied to IMAGEN data and model parameters were subsequently associated with substance use and neural connectivity. Key RDEX-ABCD parameters— including those indexing evidence accumulation for correct responses, decision threshold, and probability of go failure (i.e., attentional lapses)— were robust predictors of cigarette and cannabis use at both ages. However, parameters that described the inhibitory control process had no apparent associations with substance use. Although brain networks predicted multiple model parameters describing the go and stop process, the neural signatures of EEA, decision threshold, probability of go failure, were all associated with substance use, whereas neural signatures for the inhibitory control process (SSRT_RDEX_, SSRT_INT_) were not. Among these parameters, EEA stood out as having the most robust and consistent associations with both neural features and with substance use.

### Efficiency of evidence accumulation and substance use

EEA reflects the ability to selectively accumulate goal-relevant evidence to make adaptive decisions under conditions of background noise. Our identified associations of EEA, and its neural signature, with substance use are consistent with prior studies [19, 46, 47]. For instance, lower EEA and reductions in its neural-level correlates at ages 18–21 previously predicted greater substance use during ages 22–26 [19]. This association may reflect EEA as a domain-general decision-making mechanism, with lower levels leading to noisier and less reliable decisions [20]. It is plausible that neural systems supporting Go-trials decision making underpin real-world decisions requiring self-control, such as resisting substance use. In real-world contexts with greater uncertainty and noise, decision making is further challenged, making the accumulation of goal-relevant evidence even more difficult. Individuals with lower EEA may therefore be more prone to impulsive or maladaptive decisions that conflict with their long-term goals, ultimately increasing vulnerability to substance use.

EEA is also major driver of performance across executive tasks, including those measuring inhibition [48], and may act as a promising computationally characterized transdiagnostic vulnerability factor in psychopathology [20, 49]. Lower EEA has been proposed as a risk factor for various disorders, especially those involving impairments in cognitive control [24, 50–52]. These findings are consistent with ours in indicating that lower EEA is linked with impaired cognitive control and may reflect a common pathological process underlying diverse externalizing psychopathologies.

Brain networks predicting EEA, particularly involving the frontoparietal network, align with previous studies [24, 25]. Weigard et al. [24] demonstrated that EEA flexibly modulates attention in response to environmental demands, supported by task-positive networks like frontoparietal and dorsal attention networks, crucial for attention control and goal-directed cognition. Key regions in the frontoparietal network, such as the frontal eye field [53], and lateral intraparietal area [54] have also been shown to affect EEA. Beyond this, our CPM analysis suggests neural mechanisms of EEA supported by extensive brain regions, spanning cortex to subcortical networks.

### Decision threshold and substance use

Lower decision thresholds, reflecting reduced response caution, predicted increased cigarette and cannabis use, consistent with previous studies [22, 23, 47]. For example, Gao et al. [47] reported higher decision thresholds in nonsmokers than cigarette smokers. Drift-diffusion model analysis further linked lower response caution to increased risk choice and active gambling [55], behaviors commonly correlated with substance use [56]. This study suggests that individuals with lower response caution are more likely to engage in cigarette and cannabis use. Although the effect sizes are small by conventional standards, this is expected in adequately powered neuroimaging [57–59] and individual differences [60] studies, where effect sizes are typically modest but more reproducible than inflated estimates from underpowered work [57–59]. These reproducible associations may offer clinically meaningful insights into decision-making processes linked to substance-use risk and may have a substantial impact at the population level [61]. Moreover, our sample includes a meaningful subset of high-frequency users, and frequent use in late adolescence/early adulthood is a key risk marker for later substance use disorders [62, 63]; for example, higher age-18 substance use disorder symptoms severity predicted greater adult prescription drug use (odds ratio [OR] = 1.56), supporting relevance to emerging problematic-use trajectories and motivating replication in clinically diagnosed substance use disorder samples.

### Interpretation of SSRT results

SSRT was not as sensitive and robust as markers derived from Go trials (e.g., EEA) in neuroimaging predictions or substance use associations. A meta-analysis [64] highlighted replicability limitations of response inhibition task (e.g., Go/No-Go, SST) as markers for substance use disorder. SSRT demonstrates lower reliability over time than manifest behavior variables [65–68], whereas measures of manifest behavior, which are directly observable task performance measures (e.g., Go RT and SSD), and non-inhibitory processing tend to be more stable over time [66]. Aligned with this, EEA was the most test-retest reliable parameter across IMAGEN waves, potentially explaining its superior performance.

The relatively poor performance of SSRT likely partly stems from limited Stop trials (e.g., 60 vs. 300 Go trials), which reduces reliability for individual-differences analyses [4]. Go-trial measures like EEA are derived from more observations and thus are more stable. Although RDEX-ABCD improved SSRT prediction, limited Stop-trial data may still constrain the detection of brain–behavior associations relevant to inhibitory control deficits in psychiatric populations. Another explanation may relate to modeling differences. SSRT is typically estimated with non-parametric (integration) or parametric (ex-Gaussian) approaches. In contrast, EEA is derived from fine-grained computational components, potentially enhancing predictive validity. Future research could utilize models that describe both the go and stop processes as evidence accumulation processes, allowing for a more direct comparison. However, such models currently display problematic parameter recovery [9], limiting their research applications. Although SSRT_INT_ remains the gold standard measure of inhibitory control [4] and SSRT_RDEX_ represents a methodological advance, the lack of association with substance use in this well-powered sample casts doubt on its utility as a risk marker.

### Exploring go process insights for understanding latent stop process

The go process could provide valuable insights into the latent stop process, as evidence suggests a potential link between the two [69]. Stop and go processes share a common neural basis [70, 71]. White et al. [70] found drift rate on Go trials positively correlated with activation of inhibitory-control regions (i.e., right inferior frontal gyrus and basal ganglia) during Stop trials. Individuals with stronger go processing, reflected by drift rates, Go RT, and frontal pole activation, also demonstrated stronger inhibitory processing, as shown by SSRTs and the stopping-network activation. These findings indicated a robust relationship between stimulus-processing speed and inhibitory processing at the neural level.

## CONCLUSION

The RDEX-ABCD model was applied to IMAGEN to estimate mechanistic processes underlying SST performance. This model, in contrast to previous work (including work in this sample [30]) that relied on summary statistics, affords richer insight into the potential underlying cognitive processes engaged during the SST. We found that three go process parameters—EEA, decision threshold, and probability of go failure—as well as their neural signatures could predict cigarette and cannabis use. In comparison, stop process parameters exhibited poorer reliability, weaker relations with neural data, and no apparent predictive utility for substance use, highlighting the need for further refinement in modeling stop-related mechanisms. Notably, our findings underscore the substantial potential of EEA, other general mechanisms of decision making, and their neural correlates, as robust predictors of vulnerability to substance use.

## Supplementary Material

Supplemental Material

**Supplementary information** The online version contains supplementary material available at https://doi.org/10.1038/s41386-026-02401-6.

## Figures and Tables

**Table 1.** Demographics for computational modeling across two timepoints.

	age 19	age 23	*P* value
*N* (all)	1347	1179	
*N* (included)	1256	1089	
Age (years)	19.0 (0.7)^a^	22.6 (0.7)	
Sex (M/F)	610/646	520/569	0.724^b^
Go RT (ms)	389.9 (62.2)	395.9 (69.0)	0.861^c^
Stop RT (ms)	344.3 (56.1)	351.7 (61.7)	0.167^c^
SSD (ms)	167.2 (79.9)	168.8 (82.1)	0.499^c^
Go accuracy (%)	94.7 (4.4)	94.8 (4.7)	0.943^c^
pOmission during Go trials (%)	1.5 (3.4)	2.0 (4.1)	0.0001^d^
pCommission during Stop trials (%)	49.7 (1.8)	49.5 (1.9)	0.270^c^

These data pertain to the participants included in the behavioral analyses. N, number of participants; M/F, males/females; Go RT, reaction time on Go trials; Stop RT, reaction time on Failed Stop trials; SSD, stop signal delay; pOmisssion, probability of go omissions (no response); pCommission, probability of commission on Stop trials;

a, Mean (standard deviation), unless otherwise indicated;

b, Paired *t*-test;

c, *χ*^2^ test;

d, Wilcoxon signed-rank test.

## Data Availability

IMAGEN data are available from a dedicated database: https://imagen2.cea.fr. Due to participant consent restrictions, IMAGEN data cannot be made fully open access. Code for CPM analysis is available at https://osf.io/6ejpd/.
