# Supplementary material for: Model-based analysis of stop-signal data reveals robust neural and clinical correlates of evidence accumulation but not inhibition
Source: Neuropsychopharmacology. Author manuscript; Available in PMC 2026 Sep 21. (PMC13592875; doi:10.1038/s41386-026-02401-6)
Supplement: Supplementary file 1 [file NIHMS2208875-supplement-Supplemental_Material.docx]

**Supporting Information for**

Model-based analysis of stop-signal data reveals robust neural and clinical correlates of evidence accumulation but not inhibition

**Authors**

Yihe Weng^1^, Rory Boyle^1^, Chi Tak Lee^1^, Declan Quinn^1^, Clodagh Earley^1^, Maike Splittgerber^1^，Lili Zhang^2^, Luisa Franzen^1,3^, Tobias Banaschewski^4^, Arun L.W. Bokde^5^, Sylvane Desrivières^6^, Herta Flor^7,8^, Antoine Grigis^9^, Hugh Garavan^10^, Penny Gowland^11^, Andreas Heinz^12^, Rüdiger Brühl^13^, Jean-Luc Martinot^14^, Marie-Laure Paillère Martinot^15^, Eric Artiges^16^, Jane McGrath^5^, Frauke Nees^4,7,17^, Dimitri Papadopoulos Orfanos^9^, Luise Poustka^18^, Nathalie Holz^4^, Sarah Hohmann^4, 19^, Michael N. Smolka^20^, Nilakshi Vaidya^21^, Gunter Schumann ^20, 21^, Henrik Walter^12^, Alexander Weigard*^22^, Robert Whelan*^1^, IMAGEN Consortium

* These authors contributed equally

**Corresponding author names**: Robert Whelan

**Email:** [Robert.Whelan@tcd.ie](mailto:Robert.Whelan@tcd.ie)

**This file includes:**

Supporting text

Figures S1 to S12

Tables S1 to S9

SI References

***Methods***

### *Participants*

This study was approved by the institutional ethics committee of King’s College London (PNM/10/11-126), University of Nottingham (D/11/2007), Trinity College Dublin (SPREC092007-01), Technische Universitat Dresden (EK 235092007), Commissariat a l'Energie Atomique et aux Energies Alternatives, INSERM (2007-A00778-45), University Medical Center at the University of Hamburg (M-191/07) and in Germany at medical ethics committee of the University of Heidelberg (2007-024N-MA) in accordance with the Declaration of Helsinki.

Participants were excluded based on criteria outlined by Weigard*, et al.* [1], specifically if their behavioral data did not align with the race model assumptions. Exclusion criteria included a choice accuracy rate below 55%, an excessive omission rate (> 0.25) on Go trials, a low successful inhibition rate (< 0.25) on Stop trials, and mean RT on Stop trials being greater than mean RT on Go trials. Additionally, mean RT changes were assessed over the course of the task, and participants with substantial changes (> 0.5 s) were excluded, by fitting a linear model predicting RT by trial number (1–360). The race model cannot explain inhibition functions where the probability of responding decreases as SSDs get longer, evaluated by fitting a logistic regression model predicting the probability of responding by SSD. Participants with negative regression coefficients, indicating a decrease in responding probability as SSDs increased, were excluded.

Other exclusion criteria included the presence of a rare tracking algorithm glitch causing SSDs to remain at 0 s and SSDs > 0.7 s, which truncated the visual Stop signal presentation. Additionally, before modeling, any trials with RT < 0.15 s were excluded as fast guesses, and RT > 1.5 s were excluded as abnormally slow responses. Ultimately, 1,256 participants at age 19 and 1,089 participants at age 23 were included for RDEX-ABCD model estimation.

Perceptual growth function in RDEX-ABCD model

The perceptual growth function in RDEX-ABCD captures the relationship between SSD duration and the processing of the Go stimuli. In this function, evidence signals for the matching and mismatching accumulators on Stop trials of a given SSD are the sum of 1) a processing speed component that drives evidence accumulation for both choices equally and 2) a discrimination component that favors the choice matching the presented stimulus. Processing speed is determined by parameter *v*_0_ and is identical across all SSDs. The discrimination component is completely absent (equal to 0) at a 0-s SSDs, as the choice stimulus is not presented, but increases either in a linear or non-linear function (determined via model comparison) for both matching and mismatching components until they reach the level of *v*_t_ and *v*_f_, as determined by Go trials. Therefore, the match and mismatch rates are identical on 0s SSD trials and gradually move apart from each other at longer SSDs until they become equal to their Go trial levels.

Model evaluation

To determine whether two features of the original RDEX-ABCD model were necessary for evaluating cognitive processes in the IMAGEN dataset, four models with varying combinations of each feature were tested at each timepoint. First, given that the original model selection analysis in the ABCD baseline sample featured in Weigard, *et al.* [1] found only modest support for the inclusion of the trigger failure (*P*_tf_) parameter, the current study assessed whether model fit was improved by the inclusion of this parameter. Second, although the growth in choice evidence quality with increasing SSD appeared to be described well by a linear function in the ABCD baseline sample, we determined whether a more flexible functional form, the generalized power function, was necessary to describe the same process in the older IMAGEN sample.

### *Model estimation*

Individual-level Bayesian parameter estimation employed the differential-evolution Markov chain Monte Carlo algorithm [2] to sample from the posterior distribution. Sampling was conducted using a number of chains equivalent to three times the number of parameters in a given model (e.g., 33 chains for the 11-parameter included in the RDEX-ABCD linear model with *P*_tf_). During model estimation, the *P*_tf_ and *P*_gf_ parameters were estimated on the probit scale and subsequently transformed to their natural scale for interpretability.

Sampling was conducted using automated functions in DMC that allow for a variable burn-in period that occurs until chains have converged to a stable distribution and finishes once the Gelman-Rubin diagnostic statistic $\hat{R}$ < 1.10 [3] indicates the convergence of the differential-evolution Markov chain Monte Carlo algorithm. Additionally, visual inspections of the chains were conducted, focusing on their shapes resembling flat “fat hairy caterpillars”.

### *Parameter recovery*

Parameter recovery, a standard validity check, involves simulating data with known parameter values and re-estimating model parameters to test whether the model can recover the true parameters. We generated simulated data using the posterior medians (point estimates of a participant’s parameters) estimated from the posterior distribution of parameters from each participant. Model estimation (of the winning model from the model selection analysis) was then performed following the same steps on the simulated data as were used with the empirical data to obtain recovered parameters. The correlation between the true parameters and recovered parameters was subsequently calculated as an index of parameter recovery.

### *Exploratory factor analysis*

To explore the underlying structure of adolescent substance use, an exploratory factor analysis was performed using principal component extraction [4] on TLFB using Predictive Analytics Software (SPSS) version 20. Exploratory factor analysis explores the underlying structure of a set of observed variables without imposing a preconceived structure on the outcome. Nine items at ages 19 and 23 of TLFB were used, including alcohol, tobacco, cannabis, cocaine, ecstasy, and ketamine. The selected items can be found in [5] and Table S1. Items assessing the use of other drugs were excluded from the exploratory factor analysis due to high proportions of missing data, near-zero standard deviations, or Kaiser-Meyer-Olkin statistics below the recommended threshold of 0.5, indicating that these variables were unsuitable for factor analysis. The Kaiser-Meyer-Olkin measure of sampling adequacy was 0.81 at age 19, and 0.77 at age 23. In addition, all Bartlett's tests of sphericity were significant (age 19: χ^2^(36) = 5031.641, *P* < 0.001; age 23: χ^2^(36) = 5106.265, *P* < 0.001), indicating that there was an underlying correlation structure, and that exploratory factor analysis was appropriate. Finally, the factors were rotated using the Varimax method with Kaiser normalization to make it easier to discern the underlying measured constructs. Additional details about this data reduction step are reported in [5].

### *Elastic Net regression*

In elastic net regression, 11 RDEX-ABCD parameters and covariates (age, sex, and sites) served as predictor variables, and the TLFB-derived substance use score (e.g., Cig+CB) as targets. We applied a 10-fold nested repeated cross-validation (CV) using the *glmnet* [6] and *nestedcv* packages [7] in RStudio (version: 2023.12.1) and R (version 4.3.1). To optimize these hyperparameters, we conducted a grid search across 400 combinations of *α* and *λ* values within the nested CV framework [8]. Twenty uniformly spaced values between 0 and 1 for both *λ* and *α* were considered.

A nested CV consists of an outer loop for model performance evaluation and an inner loop for hyperparameter tune. In the outer loop, participants were allocated across 10 folds, with one fold held out for testing and the rest for training. Within each iteration of the outer loop, hyperparameter tuning was performed using another CV loop on the training data (inner loop). This inner loop further divided the training data (i.e., 9 folds from the outer loop) into 10 folds. This inner loop was carried out per hyperparameter set (e.g., *α* and *λ*) and repeated 10 times to obtain a robust estimation. The hyperparameter set yielding the best performance, with the lowest mean root mean square error (RMSE) was selected and applied to the outer loop. This process was repeated for each fold in the outer loop. The performances across these 10 models were averaged together to provide the final RMSE and R^2^ values. Lastly and independently, using the median of hyperparameters from outer CV folds, a final model was fitted on the whole data to approximate a beta value for each feature.

To ensure robust feature prediction for substance use, the 10-fold repeated nested CV process was conducted 100 times. For each feature, the mean regression coefficient (β) and the frequency of selection across iterations were calculated, and the mean RMSE and R² were used to evaluate the performance of the real model.

To assess whether the observed performance of the real model surpassed chance, random-label permutation was performed. That is, Elastic Net regression was conducted 1000 times, wherein the RDEX-ABCD model parameters were shuffled while maintaining covariates and the target variable (e.g., Cig+CB). This generated a null distribution representing the model's performance under random chance. Using this distribution, a threshold of *P* < 0.05 was established to evaluate significance at a 95% confidence level for the real model performance.

### *SSRT calculation*

Posterior samples for individuals’ SSRT_RDEX_ were computed by simulating 10,000 finishing times from ex-Gaussian distributions defined by posterior *μ*, *σ*, and *τ* , retaining SSRTs above 0.05 s, and aggregating the remaining SSRTs to form SSRT posterior distributions. In addition, an SSRT_INT_ was estimated using the integration method [9,10]. This method integrates the distribution of Go RT to identify the point at which the integral equals the probability of responding to the Stop signal. The corresponding RT at this point represents the finishing time of the stop process. SSRT_INT_ is than typically computed by subtracting SSD from the n^th^ Go RT, where the n^th^ Go RT corresponds to the

### *Generalized psychophysiological interaction analysis*

PPI is an approach for describing task-dependent FC between brain regions (details for this analysis could be found in [5]). Two separate GLMs were created for Go trials and Successful Stop trials. The Go trials model included three regressors (Go trials, Failed Stop trials, and Successful Stop trials) and 36 nuisance regressors. Given the high frequency of Go trials in SST, it was common to treat Go trials as an implicit baseline, as in previous IMAGEN studies [11,12]. Hence, a separate GLM for Successful Stop trials was built, which included two task regressors (Failed and Successful Stop trials) and 36 nuisance regressors. A gPPI analysis was conducted across the entire brain using the Shen atlas [13] for both Go and Successful Stop trials. The GLM of the Go trials included 1 regressor of another ROI eigenvariate, 3 regressors of task condition, 3 regressors of the PPI effects, and one contrast term (Equation 1). The GLM of Successful Stop trials included 1 regressor of another ROI eigenvariate, 2 regressors of task condition, 2 regressors of the PPI effects, and one contrast term (Equation 2), shown as follows:

$Y=\beta_{0}+\beta_{1}*X_{\mathrm{physio}}+\beta_{2}*X_{\mathrm{psycho}\left( \mathrm{SS} \right)}+\beta_{3}*X_{\mathrm{psycho}\left( \mathrm{FS} \right)}+\beta_{4}*X_{\mathrm{psycho}\left( \mathrm{GO} \right)}+ \beta_{5}* X_{\mathrm{physio}}*X_{\mathrm{psycho}\left( \mathrm{SS} \right)}+\beta_{6}*X_{\mathrm{physio}}*X_{\mathrm{psycho}\left( \mathrm{FS} \right)}+\beta_{7}*X_{\mathrm{physio}}*X_{\mathrm{psycho}\left( \mathrm{GO} \right)}+\varepsilon(1)$

$Y=\beta_{0}+\beta_{1}*X_{\mathrm{physio}}+\beta_{2}*X_{\mathrm{psycho}\left( \mathrm{SS} \right)}+\beta_{3}*X_{\mathrm{psycho}\left( \mathrm{FS} \right)}+ \beta_{4}* X_{\mathrm{physio}}*X_{\mathrm{psycho}\left( \mathrm{SS} \right)}+\beta_{5}*X_{\mathrm{physio}}*X_{\mathrm{psycho}\left( \mathrm{FS} \right)}+\varepsilon$(2)

Note: SS, Successful Stop trials; FS, Failed Stop trials; GO, Go trials.

Where $Y$ is the time series of seed ROI, $X_{\mathrm{physio}}$is the time series of another ROI, $X_{\mathrm{psycho}}$is the task design term, and ε is the residual term. The gPPI analysis was performed across the whole brain, and a 268 × 268 gPPI matrix was finally obtained for each participant derived from Go trials and Successful Stop trials separately.

**Sensitivity analysis**

To assess whether outliers influenced the relationship between network strength and substance use, individuals with network strength values exceeding ±3 standard deviations from the mean were excluded. Spearman correlations were then calculated between residual network strength (controlling for age, sex, site, and head motion) and residual Cig+CB scores (controlling for age, sex, and site). Results are reported in Table S8.

### *Correlation between model parameters and the intra-individual coefficient of variation (ICV)*

Our previous work [24] found a robust association between substance use and sustained attention, as indexed by the intra-individual coefficient of variation (ICV). ICV is derived from the Go trials and is computed by dividing the standard deviation of Go RT by the mean Go RT. Hence, we explored the relationship between ICV and each RDEX-ABCD model parameter, particularly those related to the go process (i.e., *v*_t_, *v_f_* or EEA). We calculated the Pearson correlation between ICV and each parameter at each timepoint separately.

***Results***

### *Parameter recovery*

Figs. S4–S5 show the parameter recovery at these ages. Notably, parameters describing the go process (*v*_t_, *v*_f_, *B*, *t*_0_), the probability of go failure (*P*_gf_: i.e., probability of “omissions” or failing to respond on a “go” trial), and the ex-Gaussian normal mean of the stop process (*mu*) exhibited robust recovery (r = 0.83-0.95 at age 19 and r = 0.85–0.95 at age 23). Other parameters (*sigma*, *tau*, *k*) showed acceptable recovery (ranging from 0.64 to 0.69 at age 19 and 0.60 to 0.68), except for one of the free parameters of the power function (*a*), which exhibited less accurate recovery (r = 0.51 at age 19 and r = 0.48 at age 23).

### *CPM predictive networks*

The connections of brain networks predicting EEA, *B and P*_gf_ at ages 19 and 23 are shown in Figs. 4A-B and Figs. S6-8. Figs. S6-8A summarize the top 10 within- and between-network connections that contributed most to these brain networks predicting EEA, *B and P*_gf_ at these ages, respectively. Specifically, the positive network predicting EEA showed connections located in the visual, motor, salience, subcortical, and default mode networks, while the negative network included connections in the frontoparietal, medial frontal, motor, salience, and default mode networks (Fig. S6). Similarly, the positive network predicting response caution (*B*) exhibited connections in the visual and salience networks, whereas the negative network involved connections in the visual, frontoparietal, medial frontal, motor, and default mode networks (Fig. S7). Finally, the positive network predicting go failure probability (*P*_gf_) showed connections in the visual, motor, subcortical, medial frontal, and default mode networks, while the negative network encompassed connections in the visual, frontoparietal, medial frontal, salience, and subcortical networks (Fig. S8). Figs. S6-8B display the degree map illustrating the number of connections from each node (top 10) in the predictive network for EEA, *B and P*_gf_.

The connections of brain for SSRT_RDEX_ and SSRT_INT_ at ages 19 and 23 are shown in Fig. S9. Figs. S10-11A summarize the top 10 within- and between-network connections that contribute most to these predictive networks predicting SSRT_RDEX_ and SSRT_INT_ at these ages, respectively. The connections in the positive network predicting SSRT_RDEX_ was located in the visual, default mode, medial frontal, and frontoparietal networks, while the connections in the negative network was located in the salience, medial frontal, frontoparietal, default mode, and visual networks (Fig. S10). Similarly, the connections in the positive network predicting SSRT_INT_ was located in the default mode, medial frontal, and frontoparietal networks, whereas the connections in the negative network was located in the visual, salience, medial frontal, frontoparietal, motor, and default mode networks (Fig. S11). Fig. S10-11B display the degree map illustrating the number of connections from each node (top 10) in the predictive network for SSRT_RDEX_.

### *Correlation between ICV and model parameters*

The correlations between model parameters and ICV were calculated at each timepoint. As shown in Table S9 and Fig. S12, all parameters, except SSRT_RDEX_ at age 23, were significantly correlated with ICV (all *P* < 0.029) at ages 19 and 23. Notably, *v*_t_ demonstrated the highest correlation with ICV (r = 0.89, *P* < 0.001) at both two timepoints.

***Discussion***

### *Enhancing SSRT prediction with computational modelling*

The RDEX-ABCD model's SSRT showed superior predictive performance compared to the integration SSRT at both ages 19 and 23, underscoring the effectiveness of computational models in enhancing neuroimaging predictions. Previous studies have indicated that computational models have significant potential to enhance statistical power [14] and refine behavioral predictions [15-17]. For instance, Wiecki et al [18] found that a drift-diffusion model analysis significantly enhanced the classification accuracy of deep brain stimulation, suggesting that computational parameters with predictive power were more easily linked with neural processes than aggregate performance scores. Findings of more significant edges (i.e., FC) selected in the predictive network predicting RDEX-ABCD SSRT – compared to integration SSRT – at age 23, indicate that SSRT estimated from the computational model may be more to sensitive differences in brain activity.

### *Absence of associations with alcohol use*

We did not find any correlation between alcohol use and RDEX-ABCD model parameters, consistent with our previous findings [5]. In our prior work, no associations were observed between alcohol use and the intra-individual coefficient of variation, an index derived from go process as well. These results may be attributed to cultural differences in social norms surrounding drinking versus smoking cannabis and cigarettes in European countries. The IMAGEN study was conducted in four European countries — the UK, Ireland, Germany, and France — where adolescent alcohol consumption is heavily influenced by social norms [19]. In these contexts, drinking is largely driven by social motives. For example, Kuntsche et al [20] found that social motives were the most prevalent and strongly linked to both drinking frequency and drunkenness among 33,813 participants aged 11–19 from 13 European countries.

In contrast, cigarette and cannabis use is more often associated with risk-taking behavior. Cannabis use, in particularly, is less socially accepted and are typically illegal. Research indicates that cannabis and cigarette use are primarily driven by coping strategies for stress and anxiety [21,22]. Lawless*, et al.* [22] identified psychosocial stress as a major risk factor for cigarette smoking, while Chabrol, *et al.* [23] found that coping motives (e.g., using marijuana to forget worries) were positively associated with marijuana use among adolescents aged 14–19. Additionally, Benschop et al. [24] linked coping motives to cannabis dependence, and personality traits like anxiety sensitivity and impulsivity are associated with cannabis use for coping [25]. Thus, for European adolescents, drinking is largely an external social behavior, while cigarette and cannabis use is more closely related to risk-taking and coping behaviors which are associated with internal or personality traits. This distinction may explain why drinking shows no significant correlation with cognitive functions measured by SST, whereas cigarette and cannabis use do.

### *Exploring go process insights for understanding latent stop process*

According to the Horse-Race model [26], both go and stop processes are engaged during Stop trials. Only a fast and efficient stop process can win the race against a fast and accurate go process [27]. Additionally, as EEA is indicated as a task-general trait [28], a higher drift rate could efficiently accumulate evidence for both Go and Stop signals, leading to a faster stop process. The SST, with more trials to estimate EEA on Go trials but fewer trials to estimate the stop process (e.g., SSRT), may allow the EEA to be measured more effectively on Go trials than on Stop trials, despite EEA being the general process that underlies performance on both types of trials.

### *Insights for future analyses of the ABCD dataset*

We conducted a thorough computational model validation, encompassing model comparison, parameter recovery, and posterior predictive checks, all of which are integral to computational modeling [29,30]. Through these rigorous procedures, we identified the optimal model among several models and achieved a robust parameter recovery particularly for key parameters of interest at both two timepoints. In addition, the high correlations observed in model parameters between ages 19 and 23 implied the consistency of cognitive processes estimated by the RDEX-ABCD model over time. These findings empower us to draw precise conclusions regarding the ability of the model to accurately capture empirical data. Although the optimal RDEX-ABCD model for 9–10-year-olds in the ABCD study incorporated parameters for trigger failure [1], this model was not optimal for IMAGEN participants (at ages 19 and 23). This discrepancy could be attributed to the maturation and development of participants in the IMAGEN study, who appear to have negligible rates of trigger failures compared with younger adolescents in the ABCD study. Furthermore, given that the released data from the ABCD study are currently from participants in early to mid-adolescence, our findings might provide valuable insights for predicting the ABCD study's future trajectory.

### *ICV and RDEX-ABCD parameters*

We found that ICV, a summary measure of response variability, was significantly correlated with most RDEX-ABCD model parameters (Table S9). There was a significant correlation (coincidentally both r = -0.54) between ICV and EEA at ages 19 and 23, consistent with a previous study [31]. The highest correlation was between *v*_t_ (Go match rate, r=-.89) and ICV, which is plausible given the theoretical framework of diffusion models. In these models, drift rate affects RT variability and accuracy, while threshold and non-decision time predominantly influence mean RT [32]). Accuracy is typically high during the Go trials in the SST, making RT variability the primary factor in estimating drift rate.

Notwithstanding the accessibility of the ICV metric (cf. computational models), the RDEX-ABCD model offers a more detailed understanding of the neurocognitive mechanisms [33] underlying behavior. Evidence accumulation models are highly biologically plausible [34], supported by single-cell recordings in primates [35-37]. Specifically, spiking activity within certain neural subpopulations during choice tasks displays properties consistent with an evidence accumulation process. For instance, both target and distractor cells in monkeys initially increase their firing rates nonselectively in the oddball discrimination task. However, as the task progresses, the firing rates become discriminative: activity in target cells either increases or remains stable, while activity in distractor cells decreases [35]. This framework provides insights into the brain’s physical implementation of these processes. Moreover, the drift rate construct is conceptually similar to sustained or selective attention, with prior studies demonstrating that less efficient evidence accumulation predicted inattention [34].

Although ICV could be significantly correlated with drift rate in tasks with ceiling accuracy, such performances are relatively rare in most tasks. When participants do not reach ceiling performance, the drift rate becomes more closely related to the accuracy than to RT variability. Similarly, in tasks of varying difficulty, the drift rate correlates with accuracy rates in more challenging tasks and with RT variability in easier tasks. Thus, ICV across different tasks may not be as robust as that of the drift rate. Drift rate estimates are advantageous because they allow for the measurement of a common construct across samples with varying abilities and tasks of different difficulty levels. Taken together, the observed similarity between ICV and drift rate arises from the drift rate's significant effect on RT variability, especially in tasks with high accuracy. The RDEX-ABCD model further enhances our understanding of the cognitive processes during Go trials by connecting these processes to performance outcomes through a mechanistic model.

Fig. S1. Distributions of substance use measures from Timeline Followback at (A) ages 19 and (B) 23. Within each panel, subplots depict past-30-day cannabis use frequency (days used), tobacco use frequency (days smoked), alcohol use frequency (days used), binge drinking frequency (days with ≥5 drinks for males/≥4 drinks for females), and the Cig+CB factor score. Cig+CB factor scores were derived from exploratory factor analysis of TLFB data and primarily reflect the tobacco and cannabis use items. The final subplot summarizes the prevalence of other drug use (cocaine/ecstasy/ketamine). At age 19, the proportion of high-frequency users (cannabis/tobacco: >20 days/month; alcohol: >10 days/month; binge drinking: >4 days/month; other drugs: any cocaine/ecstasy/ketamine use) was 2.74% (cannabis), 18.83% (tobacco), 11.33% (alcohol), 13.20% (binge drinking), and 5.05% (other drugs). At age 23, the corresponding proportions were 4.72%, 18.27%, 15.52%, 14.07%, and 9.61%, respectively.

Fig. S2. (A) The schematic of 10-fold nested cross-validation in Elastic Net regression.

(B) Performances of Elastic Net regression at age 19. (C) Performances of Elastic Net regression at age 23. The blue dash line indicates the performances of the true model. The distributions of performances for substance use were obtained from 1000 random models.

**Fig. S3. Posterior predictive checks for Stop trials at ages (A) 19 and (B) 23.** Plots comparing empirical data from Stop trials (black dots) with model posteriors’ median predictions (dark red line) and their 99% credible intervals (red shading). Plots show, from left to right, the model’s ability to account for Go choice accuracy on Stop trials of different stop-signal delays (SSDs), individuals’ inhibition functions, and median response times (RTs) across Stop trials of different SSDs. **Posterior predictive checks for Go choice response time data at age (C) 19 and (D) 23.** Defective cumulative distribution function plots that represent the cumulative probability of a “left” choice response (solid line) and “right” choice response (dashed line) over time across right arrow and left arrow stimuli and across Go choice only (GO) and Stop-signal (SS) trials. Red lines represent empirical data, and black lines represent model predictions. Points represent the .1, .3, .5, .7, and .9 quantiles of response times for each condition.

Fig. S4. Results from the parameter recovery study at age 19 were used to estimate parameters (both the initial parameter values estimated from empirical data and the parameter values recovered from simulated data). Scatterplots illustrate the relations between the simulated (“sim.”) and recovered (“rec.”) parameter values as compared to the diagonal solid line indicating perfect recovery. Numbers above each plot report the correlation coefficient (r) for each relation and the posterior coverage proportions (c) for each parameter, which indicate the proportion of data-generating parameter values that fall within the 95% posterior credible interval for the parameters recovered from the generated data.

Fig. S5. Results from the parameter recovery study at age 23 were used to estimate parameters (both the initial parameter values estimated from empirical data and the parameter values recovered from simulated data). Scatterplots illustrate the relations between the simulated (“sim.”) and recovered (“rec.”) parameter values as compared to the diagonal solid line indicating perfect recovery. Numbers above each plot report the correlation coefficient (r) for each relation and the posterior coverage proportions (c) for each parameter, which indicate the proportion of data-generating parameter values that fall within the 95% posterior credible interval for the parameters recovered from the generated data.

Fig. S6. The connectome patterns of predictive networks for efficiency of evidence accumulation (EEA). **(A)** Heatmap of network connectivity contributing to predictive network for EEA. The values of selected edges, representing connections, are aggregated to derive network strength for each cell across all participants. Mean values then are obtained by averaging network strength for each cell across participants. The heatmap only displays the top 10 network strength values. Overlapping connections between ages 19 and 23 are denoted by a star symbol (*). **(B)** Degree Map of Predictive Network for EEA. The degree map illustrates the number of connections from each node (top 10) in the predictive network for EEA. The red/blue colour represents a positive/negative network. MF, medial frontal; FP, frontoparietal; DMN, default mode; MOT, motor; VI, visual I; VII, visual II; VAs, visual association; SAL, salience; SC, subcortical; CBL, cerebellar. R/L, right/left hemisphere.

Fig. S7 The connectome patterns of predictive networks for efficiency of decision threshold (*B*). **(A)** Heatmap of network connectivity contributing to predictive network for B**.** The values of selected edges, representing connections, are aggregated to derive network strength for each cell across all participants. Mean values then are obtained by averaging network strength for each cell across participants. The heatmap only displays the top 10 network strength values. Overlapping connections between ages 19 and 23 are denoted by a star symbol (*). **(B)** Degree Map of Predictive Network for B. Degree map illustrates the number of connections from each node (top 10) in the predictive network for B. Red/blue colour represents positive/negative network. MF, medial frontal; FP, frontoparietal; DMN, default mode; MOT, motor; VI, visual I; VII, visual II; VAs, visual association; SAL, salience; SC, subcortical; CBL, cerebellar. R/L, right/left hemisphere.

Fig. S8. The connectome patterns of predictive networks for Probability of go failure (*P*_gf_). **(A)** Heatmap of *network connectivity contributing to predictive network for* P_gf_*.* The values of selected edges, representing connections, were aggregated to derive network strength for each cell across all participants. Mean values then were obtained by averaging network strength for each cell across participants. The heatmap only displays the top 10 network strength values. Overlapping connections between ages 19 and 23 are denoted by a star symbol (*). **(B)** Degree Map of Predictive Network for P_gf_. The degree map illustrates the number of connections from each node (top 10) in the predictive network for P_gf_. The red/blue color represents a positive/negative network. MF, medial frontal; FP, frontoparietal; DMN, default mode; MOT, motor; VI, visual I; VII, visual II; VAs, visual association; SAL, salience; SC, subcortical; CBL, cerebellar. R/L, right/left hemisphere.

Fig. S9. Connectome of positive and negative networks of SSRT_RDXE_ and SSRT_INT_ at two timepoints. (A) Predictive networks at age 19. (B) Predictive networks at age 23. Red and blue color represent positive and negative networks separately. MF, Medial frontal; FP, Frontoparietal; DMN, Default mode; MOT, Motor; VI, Visual I; VII, Visual II; VAs, Visual association; SAL, Salience; SC, Subcortical; CBL, Cerebellar. R/L, right/left hemisphere.

Fig. S10. The connectome patterns of predictive networks for stop signal reaction time derived from the RDEX-ABCD model (SSRT_RDEX_). **(A)** Heatmap of network connectivity contributing to predictive network for SSRT_RDEX_. The values of selected edges, representing connections, are aggregated to derive network strength for each cell across all participants. Mean values then are obtained by averaging network strength for each cell across participants. The heatmap only displays the top 10 network strength values. Overlapping connections between ages 19 and 23 are denoted by a star symbol (*). **(B)** Degree Map of Predictive Network for SSRT_RDEX_. The degree map illustrates the number of connections from each node (top 10) in the predictive network for SSRT_RDEX_. The red/blue colour represents a positive/negative network. MF, medial frontal; FP, frontoparietal; DMN, default mode; MOT, motor; VI, visual I; VII, visual II; VAs, visual association; SAL, salience; SC, subcortical; CBL, cerebellar. R/L, right/left hemisphere.

Fig. S11. The connectome patterns of predictive networks for stop signal reaction time derived from the integration method (SSRT_INT_). (**A)** Network connectivity contributing to predictive networks for SSRT_INT_. The values of selected edges, representing connections, are aggregated to derive network strength for each cell across all participants. Mean values then are obtained by averaging network strength for each cell across participants. The heatmap only displays the top 10 network strength values. Overlapping connections between ages 19 and 23 are denoted by a star symbol (*). **(B)** Degree Map of Predictive Network for SSRT_INT_. The degree map illustrates the number of connections from each node (top 10) in the predictive network for SSRT_INT_. The red/blue colour represents a positive/negative network. MF, medial frontal; FP, frontoparietal; DMN, default mode; MOT, motor; VI, visual I; VII, visual II; VAs, visual association; SAL, salience; SC, subcortical; CBL, cerebellar. R/L, right/left hemisphere.

Fig. S12. Correlation between ICV and model Parameters at ages 19 (A) and 23 (B).

**Table S1 Timeline Followback items used for the exploratory factor analysis.**

| ID | Item | Content |  |
| --- | --- | --- | --- |
| 1 | alcohol1 | Total of days using alcohol in the past 30 days |  |
| 2 | alcohol2 | Total of alcohol drink units in past 30 days |  |
| 3 | alcohol3 | Total of days using = 5 (for boys)/ = 4 (for girls) alcohol drink units in past 30 days |  |
| 4 | alcohol4 | Total cost of alcohol in past 30 days: |  |
| 5 | cannabis1 | Total of days using cannabis in the past 30 days |  |
| 6 | cocaine1 | Total of days using cocaine in the past 30 days |  |
| 7 | ecstasy1 | Total of days using ecstasy in the past 30 days |  |
| 8 | ketamine1 | Total of days using ketamine in the past 30 days |  |
| 9 | tobacco1 | Total of days smoking tobacco in the past 30 days |  |

**Table S2 Model comparison matrices for all models**

| Models | np | age 19 |  | age 23 |  |
| --- | --- | --- | --- | --- | --- |
|  |  | DIC | BPIC | DIC | BPIC |
| model 1: linear, *P*_tf_ | 10 | 4599 | 6512 | 4013 | 5705 |
| model 2: linear, no *P*_tf_ | 11 | 1303 | 1926 | 1087 | 1636 |
| model 3: power function, *P*_tf_ | 12 | 3427 | 4820 | 3060 | 4293 |
| model 4: power function, no *P*_tf_ | 11 | 0 | 0 | 0 | 0 |

Note: np, number of parameters in each model; DIC, deviance information criterion; BPIC, Bayesian predictive information criterion. *P*_tf_, trigger failure probability parameters. model 1, linear function with *P*_tf_; model 2, linear function without *P*_tf_; model 3, power function with *P*_tf_; model 4, power function without *P*_tf_. All model comparison metrics are zero-based, by subtracting the lowest value from all other values in each comparison, to improve clarity.

Table S3 RDEX-ABCD model parameters and prior information

| **Parameter** | **Definition** | **Broad prior** | |
| --- | --- | --- | --- |
|  |  | **location** | **scale** |
| *t*_0_ | Go non-decision time | 0.15 | 0.1 |
| *B* | Go decision threshold | 1 | 1 |
| *v*_t_ | Go match rate | 3 | 2 |
| *v*_f_ | Go mismatch rate | 1 | 2 |
| *v*_0_ | Go processing speed | 2 | 2 |
| *a* | Free parameter of growth function (power function) | 10 | 1 |
| *k* | Free parameter of growth function | 0 | 1 |
|  | (power function) |  |  |
| *g* | Free parameter of growth function (linear fucntion) | 3 | 2 |
| *mu* | Stop ex-Gaussian normal mean | 0.5 | 0.3 |
| *sigma* | Stop ex-Gaussian normal SD | 0.05 | 0.1 |
| *tau* | Stop ex-Gaussian exponential mean | 0.1 | 0.1 |
| *P*_gf_ | Probability of go failure | 0 | 1 |
| *P*_tf_ | Probability of trigger failure | 0 | 1 |

Table S4 RDEX-ABCD model parameters definitions and estimates.

| Parameter | Definition | age 19 | | age 23 | |
| --- | --- | --- | --- | --- | --- |
|  |  | Mean | SD | Mean | SD |
| ***t*_0_** | Go non-decision time | 0.149 | 0.032 | 0.161 | 0.032 |
| ***B*** | Go decision threshold | 1.284 | 0.297 | 1.284 | 0.297 |
| ***v*_t_** | Go match rate | 5.352 | 1.146 | 5.516 | 1.146 |
| ***v*_f_** | Go mismatch rate | 1.059 | 0.849 | 1.096 | 0.849 |
| ***v*_0_** | Go processing speed | 3.068 | 1.171 | 3.084 | 1.171 |
| ***a*** | Free parameter of growth function | 10.035 | 0.074 | 10.039 | 0.074 |
| ***k*** | Free parameter of growth function | -0.308 | 0.577 | -0.329 | 0.577 |
| ***mu*** | Stop ex-Gaussian normal mean | 0.192 | 0.044 | 0.200 | 0.044 |
| ***sigma*** | Stop ex-Gaussian normal SD | 0.034 | 0.019 | 0.032 | 0.019 |
| ***tau*** | Stop ex-Gaussian exponential mean | 0.032 | 0.016 | 0.031 | 0.016 |
| ***P*_gf_** | Probability of go failure | 0.017 | 0.035 | 0.023 | 0.035 |
| EEA | Efficiency of evidence accumulation | 4.293 | 0.952 | 4.419 | 1.016 |
| SSRT_RDEX_  (s) | Mean stop signal reaction time (Simulation) | 0.230 | 0.032 | 0.236 | 0.032 |
| SSRT_INT_  (s) | Mean stop signal reaction time (Integration) | 0.204 | 0.445 | 0.201 | 0.104 |

Note: Bold font represents the first-order parameters estimated from the RDEX-ABCD model. s, second.

Table S5 CPM performance across various cross-validation (CV) methods.

| **Condition** | **Timepoint** | **Parameters** | **10-fold CV** | | | **5-fold CV** | | | | **leave-site-out CV** | | |
| --- | --- | --- | --- | --- | --- | --- | --- | --- | --- | --- | --- | --- |
|  |  |  | **r** | **R² (%)** | ***P*** | | **r** | **R² (%)** | ***P*** | **r** | **R² (%)** | ***P*** |
| Go trials | Age 19 | EEA | **0.35** | **12.18** | **<0.001** | | **0.35** | **12.08** | **<0.001** | **0.30** | **9.16** | **<0.001** |
|  |  | *P*_go failure_ | **0.28** | **7.91** | **<0.001** | | **0.27** | **7.44** | **<0.001** | **0.24** | **5.70** | **<0.001** |
|  |  | Threshold | **0.10** | **1.09** | **0.004** | | **0.10** | **1.12** | **0.003** | **0.09** | **0.53** | **0.002** |
|  | Age 23 | EEA | **0.33** | **11.21** | **<0.001** | | **0.33** | **10.79** | **<0.001** | **0.28** | **7.74** | **<0.001** |
|  |  | *P_go_* _failure_ | **0.28** | **8.02** | **<0.001** | | **0.28** | **7.62** | **<0.001** | **0.26** | **6.89** | **<0.001** |
|  |  | Treshold | **0.14** | **2.00** | **0.001** | | **0.14** | **1.99** | **0.001** | **0.07** | **0.82** | **0.012** |
| Successful Stop | Age 19 | SSRT_RDEX_ | **0.21** | **4.28** | **<0.001** | | **0.20** | **4.10** | **<0.001** | **0.10** | **1.04** | **0.001** |
|  |  | SSRT_INT_ | **0.16** | **2.73** | **<0.001** | | **0.15** | **2.40** | **<0.001** | **0.09** | **0.76** | **0.002** |
|  | Age 23 | SSRT_RDEX_ | **0.17** | **2.93** | **<0.001** | | **0.17** | **3.05** | **<0.001** | **0.21** | **4.48** | **<0.001** |
|  |  | SSRT_INT_ | 0.07 | 0.51 | 0.077 | | **0.07** | **0.61** | **0.032** | 0.02 | 0.06 | 0.194 |

Table S6 Correlation between combined network strengths at ages 19 and 23.

| Condition | Parameter | R value | *P* value |
| --- | --- | --- | --- |
| Go trials | EEA | 0.60 | <0.001 |
|  | *B* | 0.30 | <0.001 |
|  | *P*_gf_ | 0.25 | 2.64E-12 |
| Successful Stop | SSRT_RDEX_ | 0.42 | <0.001 |
|  | SSRT_INT_ | 0.25 | 1.71E-11 |

Table S7 Correlation between Cig+CB and combined network strength (FDR correction, q < 0.05).

| Condition | Timepoint | Parameter | Rho | *P* value |  |
| --- | --- | --- | --- | --- | --- |
| Go trials | age 19 | EEA | -0.04 | 0.226 |  |
|  |  | *P*_gf_ | **0.07** | **0.016** |  |
|  |  | *B* | -0.06 | 0.059 |  |
|  | age 23 | EEA | **-0.12** | **2.57E-04** |  |
|  |  | *P*_gf_ | 0.06 | 0.058 |  |
|  |  | *B* | **-0.11** | **4.99E-04** |  |
| Successful Stop trials | age 19 | SSRT_RDEX_ | 0.01 | 0.731 |  |
|  |  | SSRT_INT_ | 0.00 | 0.990 |  |
|  | age 23 | SSRT_RDEX_ | 0.04 | 0.284 |  |
|  |  | SSRT_INT_ | -0.01 | 0.862 |  |

Table S8 Correlation between Cig+CB and combined network strength after removing ±3 SD outliers (FDR correction, q < 0.05).

| Condition | Timepoint | Parameter | Rho | *P* value |
| --- | --- | --- | --- | --- |
| Go trials | age 19 | EEA | -0.03 | 0.338 |
|  |  | *P*_gf_ | **0.07** | **0.020** |
|  |  | *B* | -0.04 | 0.204 |
|  | age 23 | EEA | **-0.11** | **0.001** |
|  |  | *P*_gf_ | 0.07 | 0.041 |
|  |  | *B* | **-0.11** | **0.001** |
| Successful Stop trials | age 19 | SSRT_RDEX_ | 0.01 | 0.631 |
|  |  | SSRT_INT_ | -0.01 | 0.710 |
|  | age 23 | SSRT_RDEX_ | 0.03 | 0.347 |
|  |  | SSRT_INT_ | -0.02 | 0.631 |

Table S9 Correlation between parameters and ICV (FDR correction, q < 0.05).

| Parameter | R value | *P* value | R value | *P* value |
| --- | --- | --- | --- | --- |
|  | age 19 |  | age 23 |  |
| EEA | **-0.54** | **<0.001** | **-0.54** | **<0.001** |
| *t*_0_ | **-0.23** | **8.07E-14** | **-0.2** | **1.38E-10** |
| *B* | **-0.25** | **4.44E-16** | **-0.18** | **6.82E-09** |
| *v*_t_ | **-0.89** | **<0.001** | **-0.89** | **<0.001** |
| v_f_ | **-0.6** | **<0.001** | **-0.6** | **<0.001** |
| *v*_0_ | **-0.25** | **4.44E-16** | **-0.29** | **<0.001** |
| *a* | **-0.26** | **<0.001** | **-0.24** | **2.33E-15** |
| *k* | **0.31** | **<0.001** | **0.32** | **<0.001** |
| *mu* | **-0.17** | **4.53E-08** | **-0.07** | **0.029** |
| *sigma* | **0.19** | **1.22E-09** | **0.14** | **4.19E-06** |
| *tau* | **0.15** | **1.27E-06** | **0.07** | **0.022** |
| *P*_gf_ | **0.53** | **<0.001** | **0.56** | **<0.001** |
| SSRT_REDX_ | **-0.1** | **0.003** | -0.02 | 0.623 |

**SI References**

1	Weigard A, Matzke D, Tanis C, Heathcote A. A cognitive process modeling framework for the ABCD study stop-signal task. Dev Cogn Neurosci. 2023;59:101191.

2	Turner BM, Sederberg PB, Brown SD, Steyvers M. A method for efficiently sampling from distributions with correlated dimensions. Psychol Methods. 2013;18(3):368-84.

3	Gelman A, Meng X-L, Stern H. Posterior predictive assessment of model fitness via realized discrepancies. Statistica Sinica. 1996;6(4):733-60.

4	Gaskin CJ, Happell B. On exploratory factor analysis: a review of recent evidence, an assessment of current practice, and recommendations for future use. Int J Nurs Stud. 2014;51(3):511-21.

5	Weng Y, Kruschwitz J, Rueda-Delgado LM, Ruddy K, Boyle R, Franzen L, et al. A robust brain network for sustained attention from adolescence to adulthood that predicts later substance use. Elife. 2024.

6	Friedman J, Hastie T, Tibshirani R, Narasimhan B, Tay K, Simon N, et al. glmnet: Lasso and elastic-net regularized generalized linear models. Astrophysics Source Code Library. 2023:ascl:2308.011.

7	Lewis MJ, Spiliopoulou A, Goldmann K, Pitzalis C, McKeigue P, Barnes MR. nestedcv: an R package for fast implementation of nested cross-validation with embedded feature selection designed for transcriptomics and high-dimensional data. Bioinform Adv. 2023;3(1):vbad048.

8	Chekroud AM, Hawrilenko M, Loho H, Bondar J, Gueorguieva R, Hasan A, et al. Illusory generalizability of clinical prediction models. Science. 2024;383(6679):164-67.

9	Verbruggen F, Chambers CD, Logan GD. Fictitious inhibitory differences: how skewness and slowing distort the estimation of stopping latencies. Psychol Sci. 2013;24(3):352-62.

10	Verbruggen F, Aron AR, Band GPH, Beste C, Bissett PG, Brockett AT, et al. A consensus guide to capturing the ability to inhibit actions and impulsive behaviors in the stop-signal task. Elife. 2019;8.

11	D'Alberto N, Chaarani B, Orr CA, Spechler PA, Albaugh MD, Allgaier N, et al. Individual differences in stop-related activity are inflated by the adaptive algorithm in the stop signal task. Hum Brain Mapp. 2018;39(8):3263-76.

12	Whelan R, Conrod PJ, Poline JB, Lourdusamy A, Banaschewski T, Barker GJ, et al. Adolescent impulsivity phenotypes characterized by distinct brain networks. Nat Neurosci. 2012;15(6):920-5.

13	Shen X, Tokoglu F, Papademetris X, Constable RT. Groupwise whole-brain parcellation from resting-state fMRI data for network node identification. Neuroimage. 2013;82:403-15.

14	Stafford T, Pirrone A, Croucher M, Krystalli A. Quantifying the benefits of using decision models with response time and accuracy data. Behav Res Methods. 2020;52(5):2142-55.

15	Sandry J, Ricker TJ. Motor speed does not impact the drift rate: a computational HDDM approach to differentiate cognitive and motor speed. Cogn Res Princ Implic. 2022;7(1):66.

16	Wild MG, Cutler RA, Bachorowski JA. Quantifying social performance: A review with implications for further work. Front Psychol. 2023;14:1124385.

17	Geana A, Barch DM, Gold JM, Carter CS, MacDonald AW, 3rd, Ragland JD, et al. Using Computational Modeling to Capture Schizophrenia-Specific Reinforcement Learning Differences and Their Implications on Patient Classification. Biol Psychiatry Cogn Neurosci Neuroimaging. 2022;7(10):1035-46.

18	Wiecki TV, Poland J, Frank MJ. Model-Based Cognitive Neuroscience Approaches to Computational Psychiatry: Clustering and Classification. Clinical Psychological Science. 2015;3(3):378-99.

19	Braker AB, Soellner R. Alcohol drinking cultures of European adolescents. Eur J Public Health. 2016;26(4):581-6.

20	Kuntsche E, Gabhainn SN, Roberts C, Windlin B, Vieno A, Bendtsen P, et al. Drinking motives and links to alcohol use in 13 European countries. J Stud Alcohol Drugs. 2014;75(3):428-37.

21	Mathew AR, Hogarth L, Leventhal AM, Cook JW, Hitsman B. Cigarette smoking and depression comorbidity: systematic review and proposed theoretical model. Addiction. 2017;112(3):401-12.

22	Lawless MH, Harrison KA, Grandits GA, Eberly LE, Allen SS. Perceived stress and smoking-related behaviors and symptomatology in male and female smokers. Addict Behav. 2015;51:80-3.

23	Chabrol H, Beck C, Laconi S. Contribution of health motive to cannabis use among high-school students. Addict Behav. 2017;64:54-56.

24	Benschop A, Liebregts N, van der Pol P, Schaap R, Buisman R, van Laar M, et al. Reliability and validity of the Marijuana Motives Measure among young adult frequent cannabis users and associations with cannabis dependence. Addict Behav. 2015;40:91-5.

25	Glodosky NC, Cuttler C. Motives Matter: Cannabis use motives moderate the associations between stress and negative affect. Addict Behav. 2020;102:106188.

26	Logan GD, Cowan WB. On the Ability to Inhibit Thought and Action - a Theory of an Act of Control. Psychol Rev. 1984;91(3):295-327.

27	Sebastian A, Forstmann BU, Matzke D. Towards a model-based cognitive neuroscience of stopping - a neuroimaging perspective. Neurosci Biobehav Rev. 2018;90:130-36.

28	Loffler C, Frischkorn GT, Hagemann D, Sadus K, Schubert AL. The common factor of executive functions measures nothing but speed of information uptake. Psychol Res. 2024;88(4):1092-114.

29	Wilson RC, Collins AG. Ten simple rules for the computational modeling of behavioral data. Elife. 2019;8.

30	Heathcote A, Brown SD, Wagenmakers E-J. An Introduction to Good Practices in Cognitive Modeling. In: Forstmann BU, Wagenmakers E-J, editors. An Introduction to Model-Based Cognitive Neuroscience. New York, NY: Springer New York; 2015. p. 25-48.

31	Epstein JN, Karalunas SL, Tamm L, Dudley JA, Lynch JD, Altaye M, et al. Examining reaction time variability on the stop-signal task in the ABCD study. J Int Neuropsychol Soc. 2023;29(5):492-502.

32	Matzke D, Wagenmakers EJ. Psychological interpretation of the ex-Gaussian and shifted Wald parameters: a diffusion model analysis. Psychon Bull Rev. 2009;16(5):798-817.

33	Turner BM, van Maanen L, Forstmann BU. Informing cognitive abstractions through neuroimaging: the neural drift diffusion model. Psychol Rev. 2015;122(2):312-36.

34	Wiker T, Pedersen ML, Ferschmann L, Beck D, Norbom LB, Dahl A, et al. Assessing the Longitudinal Associations Between Decision-Making Processes and Attention Problems in Early Adolescence. Res Child Adolesc Psychopathol. 2024;52(5):803-17.

35	Smith PL, Ratcliff R. Psychology and neurobiology of simple decisions. Trends Neurosci. 2004;27(3):161-8.

36	Hanks TD, Summerfield C. Perceptual Decision Making in Rodents, Monkeys, and Humans. Neuron. 2017;93(1):15-31.

37	Cassey PJ, Gaut G, Steyvers M, Brown SD. A generative joint model for spike trains and saccades during perceptual decision-making. Psychon Bull Rev. 2016;23(6):1757-78.
